# Learning genetic epistasis using Bayesian network scoring criteria

**DOI:** 10.1186/1471-2105-12-89

**Published:** 2011-03-31

**Authors:** Xia Jiang, Richard E Neapolitan, M Michael Barmada, Shyam Visweswaran

**Affiliations:** 1 Department of Biomedical Informatics, University of Pittsburgh, Pittsburgh, PA, USA; 2 Intelligent Systems Program, University of Pittsburgh, Pittsburgh, PA, USA; 3 Clinical and Translational Science Institute, University Pittsburgh, Pittsburgh, PA, USA; 4 Department of Human Genetics, University of Pittsburgh, Pittsburgh, PA, USA; 5 Department of Computer Science, Northeastern Illinois University, Chicago, IL, USA

## Abstract

**Background:**

Gene-gene epistatic interactions likely play an important role in the genetic basis of many common diseases. Recently, machine-learning and data mining methods have been developed for learning epistatic relationships from data. A well-known combinatorial method that has been successfully applied for detecting epistasis is *Multifactor Dimensionality Reduction *(MDR). Jiang et al. created a combinatorial epistasis learning method called *BNMBL *to learn Bayesian network (BN) epistatic models. They compared BNMBL to MDR using simulated data sets. Each of these data sets was generated from a model that associates two SNPs with a disease and includes 18 unrelated SNPs. For each data set, BNMBL and MDR were used to score all 2-SNP models, and BNMBL learned significantly more correct models. In real data sets, we ordinarily do not know the number of SNPs that influence phenotype. BNMBL may not perform as well if we also scored models containing more than two SNPs. Furthermore, a number of other BN scoring criteria have been developed. They may detect epistatic interactions even better than BNMBL.

Although BNs are a promising tool for learning epistatic relationships from data, we cannot confidently use them in this domain until we determine which scoring criteria work best or even well when we try learning the correct model without knowledge of the number of SNPs in that model.

**Results:**

We evaluated the performance of 22 BN scoring criteria using 28,000 simulated data sets and a real Alzheimer's GWAS data set. Our results were surprising in that the Bayesian scoring criterion with large values of a hyperparameter called α performed best. This score performed better than other BN scoring criteria and MDR at *recall *using simulated data sets, at detecting the hardest-to-detect models using simulated data sets, and at substantiating previous results using the real Alzheimer's data set.

**Conclusions:**

We conclude that representing epistatic interactions using BN models and scoring them using a BN scoring criterion holds promise for identifying epistatic genetic variants in data. In particular, the Bayesian scoring criterion with large values of a hyperparameter α appears more promising than a number of alternatives.

## Background

The advent of high-throughput genotyping technology has brought the promise of identifying genetic variations that underlie common diseases such as hypertension, diabetes mellitus, cancer and Alzheimer's disease. However, our knowledge of the genetic architecture of common diseases remains limited; this is in part due to the complex relationship between the genotype and the phenotype. One likely reason for this complex relationship arises from gene-gene and gene-environment interactions. So an important challenge in the analysis of high-throughput genetic data is the development of computational and statistical methods to identify gene-gene interactions. In this paper we apply Bayesian network scoring criteria to identifying gene-gene interactions from genome-wide association study (GWAS) data.

As background we review gene-gene interactions, GWAS, Bayesian networks, and modeling gene-gene interactions using Bayesian networks.

### Epistasis

In Mendelian diseases, a genetic variant at a single locus may give rise to the disease [1]. However, in many common diseases, it is likely that manifestation of the disease is due to genetic variants at multiple loci, with each locus conferring modest risk of developing the disease. For example, there is evidence that gene-gene interactions may play an important role in the genetic basis of hypertension [2], sporadic breast cancer [3], and other common diseases [4]. The interaction between two or more genes to affect a phenotype such as disease susceptibility is called *epistasis*. Biologically, epistasis likely arises from physical interactions occurring at the molecular level. Statistically, epistasis refers to an interaction between multiple loci such that the net affect on phenotype cannot be predicted by simply combining the effects of the individual loci. Often, the individual loci exhibit weak marginal effects; sometimes they may exhibit none.

The ability to identify epistasis from genomic data is important in understanding the inheritance of many common diseases. For example, studying genetic interactions in cancer is essential to further our understanding of cancer mechanisms at the genetic level. It is known that cancerous cells often develop due to mutations at multiple loci, whose joint biological effects lead to uncontrolled growth. But many cancer-associated mutations and interactions among the mutated loci remain unknown. For example, highly penetrant cancer susceptibility genes, such as BRCA1 and BRCA2, are linked to breast cancer [5]. However, only about 5 to 10 percent of breast cancer can be explained by germ-line mutations in these single genes. "Most women with a family history of breast cancer do not carry germ-line mutations in the single highly penetrant cancer susceptibility genes, yet familial clusters continue to appear with each new generation" [6]. This kind of phenomenon is not yet well understood, and undiscovered mutations or undiscovered interactions among mutations are likely responsible.

Recently, machine-learning and data mining techniques have been developed to identify epistatic interactions in genomic data. Such methods include combinatorial methods, set association analysis, genetic programming, neural networks and random forests [7]. A well-known combinatorial method is *Multifactor Dimensionality Reduction *(MDR) [3,8-10]. MDR combines two or more variables into a single variable (hence leading to dimensionality reduction); this changes the representation space of the data and facilitates the detection of nonlinear interactions among the variables. MDR has been successfully applied to detect epistatic interactions in diseases such as sporadic breast cancer [3] and type II diabetes [8], typically in data sets containing at most a few hundred genetic loci.

### GWAS

The most common genetic variation is the *single nucleotide polymorphism *(SNP) that results when a single nucleotide is replaced by another in the genomic sequence. In most cases a SNP is biallelic, that is it has only two possible values among *A *and *G *or *C *and *T *(the four DNA nucleotide bases). If the alleles are *A *and *G*, a diploid individual has the SNP genotype *AA*, *GG*, or *AG*. The less frequent (rare) allele must be present in 1% or more of the population for a site to qualify as a SNP [11]. The human genome contains many millions of SNPs. In what follows we will refer to SNPs as the loci investigated when searching for a correlation of some loci with a phenotype such as disease susceptibility.

The advent of high-throughput technologies has enabled *genome-wide association studies *(GWAS). A GWAS involves sampling in a population of individuals about 500,000 representative SNPs. Such studies provide researchers unprecedented opportunities to investigate the complex genetic basis of diseases. While the data in a GWAS have commonly been analyzed by investigating the association of each locus individually with the disease [12-16], there has been application of pathway analysis in some of these studies [15,16].

An important challenge in the analysis of genome-wide data sets is the identification of epistatic loci that interact in their association with disease. Many existing methods for epistasis learning such as combinatorial methods cannot handle a high-dimensional GWAS data set. For example, if we only investigated all 0, 1, 2, 3 and 4-SNP combinations when there are 500,000 SNPs, we would need to investigate 2.604 × 10^21 ^combinations. Researchers are just beginning to develop new approaches for learning epistatic interactions using a GWAS data set [17-24]; however, the successful analysis of epistasis using high-dimensional data sets remains an open and vital problem. Cordell [25] provides a survey of methods currently used to detect gene-gene interactions that contribute to human genetic diseases. Most GWAS studies so far have been about "agnostic" discovery. Thomas [26] suggests combining data-driven approaches with hypothesis-driven, pathway-based analysis using hierarchical modeling strategies.

### Bayesian Networks

Bayesian networks [27-33] are increasingly being used for modeling and knowledge discovery in genetics and in genomics [34-41]. A *Bayesian network *(BN) is a probabilistic model that consists of a *directed acyclic graph *(DAG) *G*, whose nodes represent random variables, and a joint probability distribution *P *that satisfies the Markov condition with *G*. We say that (*G,P*) satisfies the *Markov condition *if each node (variable) in *G *is conditionally independent of the set of all its nondescendent nodes in *G *given the set of all its parent nodes. It is a theorem [31] that (*G,P*) satisfies the Markov condition (and therefore is a BN) if and only if *P *is equal to the product of the conditional distributions of all nodes given their parents in *G*, whenever these conditional distributions exist. That is, if the set of nodes is {*X*_1_, *X*_2_,...,*X*_*n*_}, and *PA*_*i *_is the set of parent nodes of *X*_1_, then

**Figure 1:**
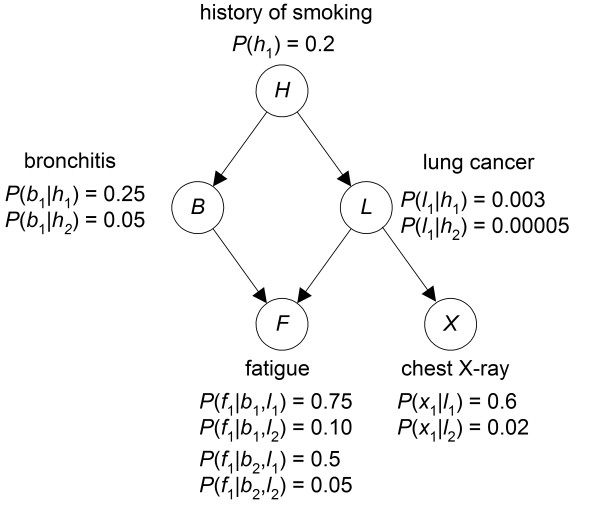
**An example BN**. A BN that models lung disorders. This BN is intentionally simple to illustrate concepts; it is not intended to be clinically complete.

BNs are often developed by first specifying a DAG that satisfies the Markov condition relative to our belief about the probability distribution, and then determining the conditional distributions for this DAG. One common way to specify the edges in the DAG is to include the edge *X*_1 _→ *X*_2 _only if *X*_1 _is a direct cause of *X*_2 _[32]. Figure 1 shows an example of a BN. A BN can be used to compute conditional probabilities of interest using a BN inference algorithm [32]. For example, we can compute the conditional probability that an individual has *lung cancer *and the conditional probability the individual has *bronchitis *given that the individual has a *history of smoking *and a positive *chest X-ray*.

Both the parameters and the structure of a BN can be learned from data. The *Data *consists of samples from some population, where each sample (called a *data item*) is a vector of values for all the random variables under consideration. Learning the structure of a BN is more challenging than learning the parameters of a specified BN structure, and a variety of techniques have been developed for structure learning. One method for structure learning, called *constraint-based*, employs statistical tests to identify DAG models that are consistent with the conditional independencies entailed by the data [42]. A second method, called *score-based*, employs heuristic search to find DAG models that maximize a desired *scoring criterion *[32]. Pierrier et al. [43] provide a detailed review of the methods for BN structure learning. Next we review scoring criteria since these criteria are the focus of this paper.

### BN Scoring Criteria

We review several BN scoring criteria for scoring DAG models in the case where all variables are discrete since this is the case for the application we will consider. BN scoring criteria can be broadly divided into Bayesian and information-theoretic scoring criteria.

#### Bayesian scoring criteria

The Bayesian scoring criteria compute the posterior probability distribution, starting from a prior probability distribution on the possible DAG models, conditional on the *Data*. For a DAG *G *containing a set of discrete random variables *V *= {*X*_1_, *X*_2_,...,*X*_*n*_} and *Data*, the following *Bayesian scoring criterion *(or simply *score*) is derived under the assumption that all DAG models are equally likely *a priori *[44,45]:(1)

where *r*_*i *_is the number of states of *X*_*i*_, *q*_*i *_is the number of different values the parents of *X*_*i *_in *G *can jointly assume, *a*_*ijk *_is the prior belief concerning the number of times *X*_*i *_took its *k*th value when the parents of *X*_*i *_took their *j*th value, and *s*_*ijk *_is the number of times in the data that *X*_*i *_took its *k*th value when the parents of *X*_*i *_took their *j*th value.

The Bayesian score given by Equation 1 assumes that our prior belief concerning each unknown parameter in each DAG model is represented by a Dirichlet distribution, where the hyperparameters *a*_*ijk *_are the parameters for this distribution. Cooper and Herskovits [44] suggest setting the value of every hyperparameter *a*_*ijk *_equal to 1, which assigns a prior uniform distribution to the value of each parameter (prior ignorance as to its value). Setting all hyperparameters to 1 yields the *K2 score *and is given by the following equation:

The K2 score does not necessarily assign the same score to Markov equivalent DAG models. Two DAGs are *Markov equivalent *if they entail the same conditional independencies. For example, the DAGs *X*→*Y *and *X *← *Y *are Markov equivalent. Heckerman et al. [45] show that if we determine the values of the hyperparameters from a single parameter *α *called the *prior equivalent sample size *then Markov equivalent DAGs obtain the same score. If we use a prior equivalent sample size *α *and want to represent a prior uniform distribution for each variable (not parameter) in the network, then for all *i*, *j*, and *k *we set *a*_*ijk *_= *α*/*r*_*i*_*q*_*i*_, where *r*_*i *_is the number of states of the *i*th variable and *q*_*i *_is the number of different values the parents of *X*_*i *_can jointly assume. When we use a prior equivalent sample size *α *in the Bayesian score, the score is called the *Bayesian Dirichlet equivalent (BDe) scoring criterion*. When we also represent a prior uniform distribution for each variable, the score is called the *Bayesian Dirichlet equivalent uniform *(*BDeu*) *scoring criterion *and is given by the following equation:

The Bayesian score does not explicitly include a *DAG penalty *for network complexity. However, a DAG penalty is implicitly determined by the hyperparameters *a*_*ijk*_. Silander et al. [46] show that if we use the BDeu score, then the DAG penalty decreases as *α *increases. The K2 score uses hyperparameters in a way that can be related to a prior equivalent sample size. When a node is modeled as having more parents, the K2 score effectively assigns a higher prior equivalent sample size to that node, which in turn decreases its DAG penalty.

#### Minimum description length scoring criteria

The *Minimum Description Length *(MDL) Principle is an information-theoretic principle [47] which states that the best model is one that minimizes the sum of the encoding lengths of the data and the model itself. To apply this principle to scoring DAG models, we must determine the number of bits needed to encode a DAG *G *and the number of bits needed to encode the data given the DAG. Suzuki [48] developed the following *MDL scoring criterion*:(2)

where *n *is the number of nodes in *G*, *d*_*i *_is the number of parameters needed to represent the conditional probability distributions associated with the *i*th node in *G*, *m *is the number of data items, *r*_*i *_is the number of states of *X*_*i*_, *x*_*ik *_is the *k*th state of *X*_*i*_, *q*_*i *_is the number of different values the parents of *X*_*i *_can jointly assume, *pa*_*ij *_is the *j*th value of the parents of *X*_*i*_, and the probabilities are estimated from the *Data*. In Equation 2 the first sum is the DAG penalty, which is the number of bits sufficient to encode the DAG model, and the second term is the number of bits sufficient to encode the *Data *given the model.

Other MDL scores assign different DAG penalties and therefore differ in the first term in Equation 2, but encode the data the same. For example, the *Akaike Information Criterion *(*AIC*) *score *is an MDL scoring criterion that uses  as the DAG penalty. We will call this score *score*_*AIC*_. In the DDAG Model section (acronym DDAG is defined in that section) we give an MDL score designed specifically for scoring BNs representing epistatic interactions.

#### Minimum message length scoring criterion

Another score based on information theory is the *Minimum Message Length Score *(*MML*) that is described in [30]. In the case of discrete variables it is equal to

where *d*_*i *_is the number of parameters stored for the *i*th node in *G *and *score*_*k*__2 _is the K2 score mentioned previously.

To learn a DAG model from data, we can score all DAG models using one of the scores just discussed and then choose the highest scoring model. However, when the number of variables is not small, the number of candidate DAGs is forbiddingly large. Moreover, the BN structure learning problem has been shown to be NP-hard [49]. So heuristic algorithms have been developed to search over the space of DAGs during learning [32].

In the large sample limit, all the scoring criteria favor a model that most succinctly represents the generative distribution. However, for practical sized data sets, the results can be quite disparate. Silander et al. [46] provide a number of examples of learning models from various data sets showing that the choice of *α *in the BDeu scoring criterion can greatly affect how many edges exist in the selected model. For example, in the case of their Yeast data set (which contains 9 variables and 1484 data items), the number of edges in the selected model ranged from 0 to 36 as the value of *α *in the Bayesian scores ranged from 2 × 10^-20 ^to 34,000. Although researchers have recommended various ways for choosing *α *and sometimes argued for the choice on philosophical/intuitive grounds [32], there is no agreed upon choice.

### Detecting Epistasis Using BNs

BNs have been applied to learning epistatic interactions from GWAS data sets. Han et al. [50] developed a Markov blanket-based method that uses a G^2 ^test instead of a BN scoring criterion. Verzilli et al. [51] represent the relationships among SNPs and a phenotype using a *Markov network *(*MN*), which is similar to a BN but contains undirected edges. They then use MCMC to do approximate model averaging to learn whether a particular edge is present. Both these methods model the relationships among SNPs besides the relationship between SNPs and a phenotype.

Jiang et al. [52] took a different approach. Since we are only concerned with discovering SNP-phenotype relationships, they used specialized BNs called DDAGs to model these relationships. DDAGs are discussed in the DDAG Model subsection of the Results section. They developed a combinatorial epistasis learning method called BNMBL that uses an MDL scoring criterion for scoring DDAGs. They compared BNMBL to MDR using the data sets developed in [10]. Each of these data sets was generated from a model that associates two SNPs with a disease and includes 18 unrelated SNPs. For each data set, BNMBL and MDR were used to score all 2-SNP models, and BNMBL learned significantly more correct models. In another study, Visweswaran et al. [53] employed a K2-based scoring criterion for scoring these same DAG models that also outperformed MDR.

In real data sets, we ordinarily do not know the number of SNPs that influence phenotype. BNMBL may not perform as well if we also scored models containing more than two SNPs. Although BNs are a promising tool for learning epistatic relationships from data, we cannot confidently use them in this domain until we determine which scoring criteria work best or even well when we try learning the correct model without knowledge of the number of SNPs in that model. We provide results of experiments investigating this performance in the Results section.

### Diagnostic BNs Containing SNP Variables

BN diagnostic systems that contain SNP information have also been learned from data. For example, Sebastiani et al. [54] learned a BN that predicts stroke in individuals with sickle cell anemia, while Meng et al. [55] learned a BN that predicts rheumatoid arthritis. In these studies candidate SNPs were identified based on known metabolic pathways. This is in contrast to the *agnostic *search ordinarily used to analyze GWAS data sets (discussed above). For example, Sebastiani et al. [54] identified 80 candidate genes and analyzed 108 SNPs in these genes.

## Results

We first describe the BN model used to model SNP interactions associated with disease. Next, we develop a BN score tailored to this model and list the other BN scores that are evaluated. Finally, we provide the results of experiments that evaluate the various BN scores and MDR using simulated data and a real GWAS data set.

### The DDAG Model

**Figure 2:**
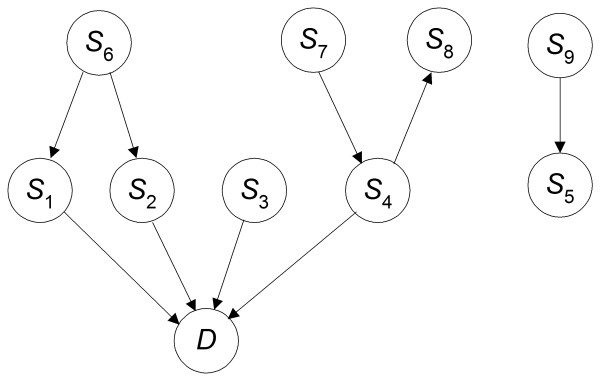
**An example DAG**. A DAG showing probabilistic relationships among SNPs and a disease *D*.

**Figure 3:**
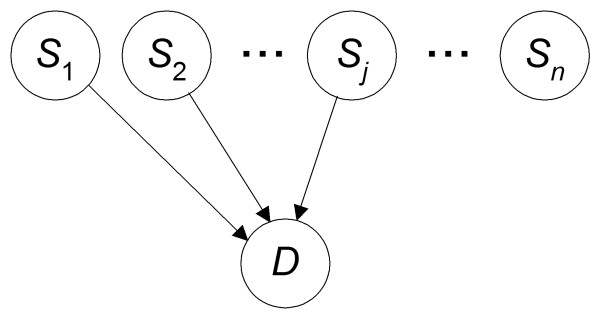
**An example DDAG**. A DDAG showing probabilistic relationships between SNPs and a disease *D*. A DDAG differs from the DAG in Figure 2 in that the relationships among the SNPs are not represented.

We use BNs to model the relationships among SNPs and a phenotype such as disease susceptibility. Given a set of SNPs {*S*_1_, *S*_2_, ...,*S*_*n*_} and a disease *D*, we consider all DAGs in which node *D *has only incoming edges and no outgoing edges. Such DAGs have the causal interpretation that SNPs are either direct or indirect causes of disease. An example of a DAG for 9 SNPs is shown in Figure 2. This DAG does not represent the relationships among gene expression levels. Rather it represents the statistical dependencies involving the disease status and the alleles of the SNPs. Since we are only concerned with modeling the dependence of the disease on the SNPs and not the relationships among the SNPs, there is no need for edges between SNPs. So we need only consider DAGs where the only edges are ones to *D*. An example of such a DAG is shown in Figure 3. We call such a model a *direct DAG *(*DDAG*).

The number of DAGs that can be constructed is forbiddingly large when the number of nodes is not small. For example, there are ~4.2 × 10^18 ^possible DAGs for a domain with ten variables [56]. The space of DDAGs is much smaller: there are 2^*n *^DDAGs, where *n *is the number of SNPs. So if we have ten SNPs, there are only 2^10 ^DDAGs. Though the model space of DDAGs is much smaller that the space of DAGs, it still remains exponential in the number of variables. In the studies reported here, we search in the space of DDAGs.

### The BN Minimum Bit Length (BNMBL) Score

An MDL score called BNMBL that is adapted to DDAGs is developed next. Each parameter (conditional probability) in a DAG model learned from data is a fraction with precision 1/*m*, where *m *is the number of data items. Therefore, it requires *O*(log_2 _*m*) bits to store each parameter. However, as explained in [57], the high order bits are not very useful. So we need use only  bits and we arrive at the DAG penalty in Equation 2.

Suppose that *k *SNPs have edges into *D *in a given DDAG. Since each SNP has three possible values, there are 3^*k *^joint states of the parents of *D*. The expected value of the number of data items, whose values for these *k *SNPs are the values in each joint state, is *m*/3^*k*^. If we approximate the precision for each of *D*'s parameters by this average, the penalty for each of these parameters is . Since the penalty for each parameter in a parent SNP is , the total DAG penalty for a DDAG model is(3)

The multiplier 2 appears in the second term because each SNP has three values. We need store only two of the three parameters corresponding to the SNP states, since the value of the remaining parameter is uniquely determined given the other two. No multiplier appears in the first term because the disease node has only two values. When we use this DAG penalty in an MDL score (Equation 2), we call the score *score*_*Epi*_.

### BN Scoring Criteria Evaluated

We evaluated the performance of MDR; three MDL scores: *score*_*Epi*_, *score*_*Suz*_, and *score*_*AIC*_; two Bayesian scores: *score*_*K*__2_, and *score*_*α*_; and the information-theoretic score *score*_*MML*_. For *score*_*α *_we performed a sensitivity analysis over the following values of *α *= 1 3, 6, 9, 12, 15, 18, 21, 24, 30, 54, 162. We evaluated two versions of each of the MDL scores. In the first version, all *n *SNPs in the domain are included in the model, though only *k *of them directly influence *D *and hence have edges to *D *in the DDAG. In this case the contribution of the SNP nodes to the DAG penalty is not included in the score because it is the same for all models. We call this version 1, and denote the score with the subscript 1 (*score*_*Epi*__1_). In the second version, only the *k *SNPs that have edges to *D *are included in the model and the remaining *n*-*k *SNPs are excluded from the model. In this case, the contributions of the *k *SNP nodes to the penalty are included because models with different values of *k *have different penalties. We call this version 2, and denote the score with the subscript 2 (e.g., *score*_*Epi*__2_). The penalty term for *score*_*Epi *_that is given in Equation 3 is for version 2.

After describing the results obtained using simulated data, we show those for real data.

### Simulated Data Results

We evaluated the scoring criteria using simulated data sets that were developed from 70 genetic models with different heritabilities, minor allele frequencies and penetrance values. Each model consists of a probabilistic relationship in which 2 SNPs combined are correlated with the disease, but neither SNP is individually correlated. Each data set has sample size equal to 200, 400, 800, or 1600, and there are 7000 data sets of each size. More details of the datasets are given in the Methods section.

For each of the simulated data sets, we scored all 1-SNP, 2-SNP, 3-SNP, and 4-SNP DDAGs. The total number of DDAGs scored for each data set was therefore 6195. Since in a real setting we would not know the number of SNPs in the model generating the data, all models were treated equally in the learning process; that is, no preference was given to 2-SNP models.

We say that a method *correctly learns *the model generating the data if it scores the DDAG representing the generating model highest out of all 6195 models. Table 1 shows the number of times out of 7000 data sets that each BN scoring criterion correctly learned the generating model for each sample size. In this table, the scoring criteria are listed in descending order according to the total number of times the correct model was learned. Table 1 shows a number of interesting results. First, the AIC score performed reasonably well on small sample sizes, but its performances degraded at larger sample sizes. Unlike the other BN scores, the DAG penalty in the AIC score does not increase with the sample size. Second, the K2 score did not perform well, particularly at small sample sizes. However, the MML1 score, which can be interpreted as the K2 score with an added DAG penalty, performed much better. This indicates that the DAG penalty in the K2 score may be too small and the increased penalty assigned by the MML1 score is warranted. Third, MDR performed well overall but substantially worse than the best performing scores. Fourth, the best results were obtained with the BDeu score at moderate values of *α*. However, the results were very poor for large values of *α*, which assign very small DAG penalties.

**Table 1:** Accuracies of scoring criteria

	Scoring Criterion	200	400	800	1600	Total
1	*score*_*α *__= 15_	4379	5426	6105	6614	22524

2	*score*_*α *__= 12_	4438	5421	6070	6590	22519

3	*score*_*α *__= 18_	4227	5389	6095	6625	22336

4	*score*_*α *__= 9_	4419	5349	5996	6546	22313

5	*score*_*α *__= 21_	3989	5286	6060	6602	21934

6	*score*_*α *__= 6_	4220	5165	5874	6442	21701

7	*score*_*MML*__1_	4049	5111	5881	6463	21504

8	*score*_*α *__= 24_	3749	5156	5991	6562	21448

9	*score*_*MDR*_	4112	4954	5555	5982	20603

10	*score*_*α *__= 3_	3839	4814	5629	6277	20559

11	*score*_*Epi*__2_	3571	4791	5648	6297	20307

12	*score*_*α *__= 30_	3285	4779	5755	6415	20234

13	*score*_*MML*__2_	3768	4914	5754	5780	20216

14	*score*_*Epi*__1_	2344	5225	6065	6553	20187

15	*score*_*Suz*__1_	3489	4580	5521	6215	19805

16	*score*_*α *__= 36_	2810	4393	5464	6150	18817

17	*score*_*α *__= 42_	2310	4052	5158	5895	17415

18	*score*_*K*__2_	1850	3475	5095	6116	16536

19	*score*_*Suz*__2_	2245	3529	4684	5673	16131

20	*score*_*α *__= 54_	1651	3297	4492	5329	14769

21	*score*_*AIC*__2_	3364	3153	2812	2520	11847

22	*score*_*AIC*__1_	2497	1967	1462	1126	7052

23	*score*_*α *__= 162_	26	476	1300	2046	3848

The number of times out of 7000 data sets that each scoring criterion identified the correct model for sample sizes of 200, 400, 800, and 1600. The last column gives the total accuracy over all sample sizes. The scoring criteria are listed in descending order of total accuracy.

The ability of the highest ranking score (the BDeu *score*_*α = *15_) to identify the correct model was compared to that of the next six highest ranking scores using the McNemar chi-square test (see Table 2). In a fairly small interval around α = 15 there is not a significant difference in performance. However, as we move away from α = 15 the significance becomes dramatic, as is the significance relative to the highest scoring non-BDeu score (*score*_*MML*__1_).

**Table 2:** Statistical comparison of accuracies of scoring criteria

	Scoring Criterion	*p*-value
1	*score*_*α *__= 15_	NA

2	*score*_*α *__= 12_	0.996

3	*score*_*α *__= 18_	0.076

4	*score*_*α *__= 9_	0.046

5	*score*_*α *__= 21_	4.086 × 10^-8^

6	*score*_*α *__= 6_	3.468 × 10^-14^

7	*score*_*MML*__1_	1.200 × 10^-20^

*P*-values obtained by comparing the accuracy of the highest ranking scoring criterion (*score*_*α *= 15_) with the next six highest ranking scoring criteria using the McNemar chi-square test. Each *p*-value is obtained by comparing the accuracies for 28,000 data sets.

BDeu scores with values of *α *in the range 12 - 18 performed significantly better than all other scores. If our goal is only to find a score that most often scores the correct model highest on low-dimensional simulated data sets like the ones analyzed here, then our results support the use of these BDeu scores. However, in practice, we are interested in the discovery of promising SNP-disease associations that may be investigated for biological plausibility. So perhaps more relevant than whether the correct model scores the highest is the *recall *of the correct model relative to the highest scoring model. The recall is given by:

where *S *is the set of SNPs in the correct model, *T *is the set of SNPs in the highest scoring model, and # returns the number of items in a set. The value of the recall is 0 if and only if the two sets do not intersect, while it is 1 if and only if all the SNPs in the correct model are in the highest scoring model. Therefore, recall is a measure of how well the SNPs in the correct model were are discovered. Recall does not measure, however, the extent to which the highest scoring model has additional SNPs that are not in the correct model (i.e., false positives).

Table 3 shows the recall for the various scoring criteria. The criteria are listed in descending order of total recall. Overall, these results are the reverse of those in Table 1. The BDeu scores with large values of α and the AIC scores appear at the top of the list. Part of the explanation for this is that these BDeu scores and AIC scores incorporate small DAG penalties, which results in larger models often scoring higher. A larger model has a greater chance of containing the two interacting SNPs. Not surprisingly *score*_*Suz*__1 _and *score*_*Suz*__2_, which have the largest DAG penalties of the MDL scores, appear at the bottom of the list. MDR again performed well but substantially worse than the best performing scores.

**Table 3:** Recall for scoring criteria

	Scoring Criterion	200	400	800	1600	Total
1	*score*_*α *= 162_	5259	6043	6566	6890	24758

2	*score*_*AIC*__2_	5204	5969	6511	6849	24533

3	*score*_*AIC*__1_	5186	5960	6481	6830	24457

4	*score*_*α *= 54_	5223	5941	6473	6813	24450

5	*score*_*K*__2_	5303	5962	6371	6747	24383

6	*score*_*α *= 42_	5203	5902	6425	6794	24324

7	*score*_*α *= 36_	5181	5866	6395	6768	24210

8	*score*_*α *= 30_	5147	5816	6352	6754	24069

9	*score*_*α *= 24_	5080	5767	6300	6725	23872

10	*score*_*α *= 21_	5031	5733	6265	6704	23733

11	*score*_*MDR*_	4870	5710	6324	6748	23652

12	*score*_*α *= 18_	4973	5681	6230	6681	23565

13	*score*_*α *= 15_	4902	5622	6183	6647	23354

14	*score*_*Epi*__1_	4984	5529	6105	6575	23193

15	*score*_*α *= 12_	4786	5531	6119	6605	23041

16	*score*_*α *= 9_	4649	5416	6026	6547	22638

17	*score*_*α *= 6_	4383	5219	5901	6453	21956

18	*score*_*MML*__1_	4151	5159	5903	6473	21686

19	*score*_*MML*__2_	3881	4969	5780	6412	21042

20	*score*_*Epi*__2_	3895	4901	5715	6329	20840

21	*score*_*α *= 3_	3953	4862	5652	6285	20752

22	*score*_*Suz*__1_	3618	4696	5595	6251	20160

23	*score*_*Suz*__2_	2500	3712	4811	5737	17760

The sum of the recall for each scoring criterion over 7000 data sets for sample sizes of 200, 400, 800, and 1600. The last column gives the total recall over all sample sizes. The scoring criteria are listed in descending order of total recall.

Perhaps the smaller DAG penalty is not the only reason that the BDeu scores with larger values of α performed best. It is possible that the BDeu scores with larger values of α can better detect the interacting SNPs than the BDeu scores with smaller values, but that the scores with larger values do poorly at scoring the correct model (the one with only the two interacting SNPs) highest because they too often pick a larger model containing those SNPs. To investigate this possibility, we investigated how well the scores discovered models 55-59 (See Supplementary Table one to [10]). These models have the weakest broad-sense heritability (0.01) and a minor allele frequency of 0.2, and are therefore the most difficult to detect.

Table 4 shows the number of times the correct hard-to-detect model scored highest for a representative set of the scores. Table 5 shows the *p*-values obtained when the highest ranking score (BDeu *score*_*α *= 54_) is compared to the next five highest ranking scores using the McNemar chi-square test. The BDeu score with large values of α performed significantly better than all other scores.

**Table 4:** Accuracies of scoring criteria on most difficult models

	Scoring Criterion	200	400	800	1600	Total
1	*score*_*α *= 54_	14	48	167	352	581

2	*score*_*α *= 162_	1	21	146	355	563

3	*score*_*α *= 36_	13	46	155	318	532

4	*score*_*α *= 21_	12	43	106	289	450

5	*score*_*α *= 18_	11	37	91	274	413

6	*score*_*MDR*_	3	25	79	245	352

7	*score*_*α *= 12_	7	25	65	215	312

8	*score*_*AIC*__2_	16	33	80	138	267

9	*score*_*α *= 9_	5	20	48	186	259

10	*Score*_*Epi*__1_	4	16	47	179	246

11	*score*_*MML*__1_	2	7	23	140	172

12	*score*_*α *= 3_	3	6	13	86	108

13	*score*_*Epi*__2_	0	1	4	72	77

14	*score*_*Suz*__1_	0	1	2	41	44

The number of times out of 500 that each scoring criterion correctly learned the correct model in the case of the most difficult models (55-59) for sample sizes of 200, 400, 800, and 1600. The last column gives the total accuracy over all sample sizes. The scoring criteria are listed in descending order of accuracy.

**Table 5:** Statistical comparison of accuracies of scoring criteria on most difficult models

Scoring Criterion	*p*-value
*score*_*α *= 54_	NA

*score*_*α *= 162_	0.610

*score*_*α *= 36_	0.147

*score*_*α *= 21_	4.870 × 10^-5^

*score*_*α *= 18_	1.080 × 10^-7^

*score*_*MDR*_	7.254 × 10^-14^

*P*-values obtained by comparing the accuracy of the highest ranking scoring criterion (*score*_*α *= 15_) with the next five highest ranking scoring criteria using the McNemar chi-square test. Each *p*-value is obtained by comparing the accuracies for 2,000 data sets generated by the hardest-to-detect models.

The BDeu scores with large α values discovered the difficult models best, though they perform poorly on the average when all models were considered. An explanation for this phenomenon is that these scores can indeed find interacting SNPs better than scores with smaller values of α. However, when the interacting SNPs are fairly easy to identify, their larger DAG penalties makes it harder for them to identify the correct model relative to other scores. On the other hand, when the SNPs are hard to detect, their better detection capability more than compensates for their increased DAG penalty. Additional file 1 provides an illustrative example of this phenomenon. *We hypothesize therefore that BDeu scores with larger values of α can better indentify interacting SNPs, even if they sometimes include extra SNPs in the highest scoring model.*

### GWAS Data Results

We evaluated the scoring criteria using a late onset Alzheimer's disease (LOAD) GWAS data set. LOAD is the most common form of dementia in the above 65-year-old age group. It is a progressive neurodegenerative disease that affects memory, thinking, and behavior. The only genetic risk factor for LOAD that has been consistently replicated involves the apolipoprotein E (APOE) gene. The ε4 APOE genotype increases the risk of development of LOAD, while the ε2 genotype is believed to have a protective effect.

The LOAD GWAS data set that we analyzed was collected and analyzed by Rieman et al. [16]. The data set contains records on 1411 participants (861 had LOAD and 550 did not), and consists of data on 312,316 SNPs and one binary genetic attribute representing the apolipoprotein E (APOE) gene carrier status. The original investigators found that SNPs on the GRB-associated binding protein 2 (GAB2) gene interacted with the APOE gene to determine the risk of developing LOAD. More details of this dataset are given in the Methods section.

To analyze this Alzheimer GWAS data set, for a representative subset of the scores listed in Table 1 we did the following. We pre-processed the data set by scoring all models in which APOE and one of the 312,316 SNPs are each parents of the disease node LOAD. The SNPs from the top 100 highest-scoring models were selected along with APOE. Using these 101 loci, we then scored all 1, 2, 3, and 4 parent models making a total of 4,254,726 models scored. We judged the effectiveness of each score according to how well it replicated the results obtained by the original investigators in [16] that the GAB2 gene is associated with LOAD. We did this by determining how many of the score's 25 highest-scoring models contained a GAB2 SNP. Table 6 shows the results. The number in each cell in Table 6 is the number of SNPs in the model, and the letter G appears to the right of that number if a GAB2 SNP appears in the model. The second to the last row in the table shows the total number of models in the top 25 that contain a GAB2 SNP. The last row in the table shows the total number of different GAB2 SNPs appearing in the top 25 models.

**Table 6:** Evaluation of scoring criteria concerning detection of GAB2 SNPs

Rank	α = 3	α = 12	α = 21	α = 54	α = 162	α = 1000	K2	MML1	MDLn	Suz1	Epi2	MDR
1	4	4	4	4 G	4 G	4	4 G	4 G	4 G	3	4 G	4

2	4	4	4	4 G	4 G	4	4 G	4 G	4 G	3 G	4 G	4

3	4	4	4 G	4 G	4	4	4 G	4 G	4 G	3 G	4 G	4

4	4	4	4	4 G	4 G	4	4	4	4 G	3	4 G	4

5	4	4	4	4	4	4	4	3	4 G	3	3	4

6	4	4	4	4	4 G	4	4	4	4	3 G	4 G	4 G

7	4	4	4	4 G	4	4	4	4	4	3 G	4	4

8	4	4	4	4	4	4	4	4	4 G	3 G	4	4 G

9	4	4	4	4 G	4	4	4	4	4 G	3 G	4 G	4

10	4	4	4	4 G	4 G	4	4	3 G	4	2	4 G	4 G

11	4	4	4 G	4 G	4 G	4 G	4 G	4	4	3	4	4

12	4	4 G	4 G	4	4 G	4 G	4 G	4	4	3 G	4	4 G

13	4	4	4	4 G	4 G	4 G	4	4 G	4 G	3 G	4	4

14	4	4	4	4	4 G	4	4 G	4 G	4	3	3 G	4

15	4	4	4 G	4 G	4 G	4	4 G	3 G	4	3 G	4 G	4 G

16	4	4	4 G	4 G	4 G	4 G	4 G	4	4	3 G	3 G	4

17	4	4	4	4 G	4 G	4	4 G	3	4 G	3	4	4 G

18	4	4	4 G	4 G	4 G	4 G	4	4 G	4 G	3 G	4	4

19	4	4	4 G	4 G	4 G	4 G	4	4 G	4 G	3 G	4	4

20	4	4	4	4 G	4 G	4 G	4	4 G	4	3 G	4 G	4 G

21	4	4	4	4 G	4	4 G	4	4 G	4 G	3 G	4 G	4 G

22	4	4 G	4	4 G	4 G	4 G	4	4	4 G	3	4 G	4

23	4	4 G	4	4 G	4	4 G	4 G	4	4 G	3 G	4	4

24	4	4	4 G	4 G	4 G	4	4	4	4 G	3 G	4 G	4

25	4	4	4	4	4 G	4	4	4	4 G	3 G	3	4

Total # G G##GGG	0	3	7	19	18	10	10	11	16	17	14	8

# Diff G	0	2	3	7	6	4	4	4	8	8	8	6

Information about the 25 highest scoring models for a variety of scoring criteria. The number on the left in a cell is the number of SNPs in the model, and the letter G appears to the right of that number if a GAB2 SNP appears in the model. The second to the last row shows the total number of models in the top 25 that contained a GAB2 SNP. The last row shows the total number of different GAB2 SNPs appearing in the top 25 models.

We included two new scores in this analysis. The first score is the BDeu score with α = 1000. We did this to test whether we can get good recall with arbitrarily high values of α. The second new score is an MDL score with no DAG penalty (labelled MDLn in the table). We did this to investigate the recall for the MDL score when we constrain the highest scoring model to be one containing four parent loci.

These results substantiate our hypothesis that larger values of α (54 and 162) can better detect the interacting SNPs. For each of the BDeu scores, the 25 highest-scoring models each contain 4 parent loci. However, when α equals 54 or 162, 19 and 18 respectively of the 25 highest-scoring models contain a GAB2 SNP, whereas for α equal to 12 only 7 of them contain a GAB2 SNP, and for α equal to 3 none of them do. The results for α equal to 1000 are not very good, indicating that we cannot obtain good results for arbitrarily large values of α. The MDL scores (MDLn, Suz1 and Epi2) all performed well, with the Suz1 score never selecting a model with more than 3 parent loci. This result indicates that the larger DAG penalty seems to have helped us hone in on the interacting SNPs. All the MDL scores detected the highest number of different GAB2 SNPs, namely 8. In comparison, MDR did not perform very well, having only 8 models of the top 25 containing GAB2 SNPs and none of the top 5 containing GAB2 SNPs.

## Discussion

We compared the performance of a number of BN scoring criteria when identifying interacting SNPs from simulated genetic data sets. Each data set contained 20 SNPs with two interacting SNPs and was generated from one of 70 different epistasis models. Jiang et al. [52] analyzed these same data sets using the BNMBL method and MDR (both of these methods are discussed in the Background section). However, that paper only investigated models with two interacting SNPs. So the 1-SNP, 3-SNP, and 4-SNP models were not competing and the learned model was restricted to be a 2-SNP model. In real applications we rarely would know how many SNPs are interacting. So this type of analysis is not as realistic as the one reported here.

Table 1 shows that the BDeu score with values of α between 12 and 18 was best at learning the correct model over all 28,000 simulated data sets. However, Table 3 shows that the BDeu score with large values of α (54 and 162) performed better at recall over all 28,000 data sets. Table 4 shows that these large values of α yield better detection of the models that are hardest to detect.

We evaluated the performance of a subset of the BN scores used in the simulated data analysis on a LOAD GWAS data set. The effectiveness of each score was judged according to how well it substantiated the previously obtained result that the GAB2 gene is associated with LOAD. As shown in Table 6, we obtained the best results with the BDeu score with large values of α. The various MDL scores also performed well.

Overall, our results are mixed. Although scores with moderate values of α performed better at actually scoring the correct model highest using simulated data sets, scores with larger values of α performed better at recall, at detecting models that are hardest to detect, and at substantiating previous results using a real data set. Our main goal is to develop a method that can discover SNPs associated with a disease from real data. Therefore, based on the results reported here, it seems that it is more promising to use the BDeu score with large values of α (54-162), rather than smaller values.

The MDL scores also performed well in the case of the real data set. An explanation for their poor performance with the simulated data sets is that their DAG penalties are either too large or too small. If we simply used an MDL score with no DAG penalty we should be able to discover interacting SNPs well (as indicated by Table 6). Once we determine candidate interactions using these scores, we can perform further data analysis of the interactions and also investigate the biological plausibility of the genotype-phenotype relationships. However, additional research is needed to further investigate a DAG penalty appropriate to this domain.

Another consideration which was not investigated here is the possible increase in false positives with increased detection capability. That is, although the BDeu score with large values of α performed best at recall and at identifying hard-to-detect models, perhaps these scores may also score some incorrect models higher, and at a given threshold might have more false positives. Further research is needed to investigate this matter.

Additional file 1 provides an illustrative example and some theoretical justification as to why a BDeu score with large values of α should perform well at discovering hard-to-detect SNP-phenotype relationships. However, further research, both of a theoretical and empirical nature, is needed to investigate the pattern of results reported here. In particular, additional simulated data sets containing data on a large number of SNPs (numbers appearing in real studies) should be analyzed to see if the BDeu score with large values of α or some other approach performs better in this more realistic setting.

## Conclusions

Our results indicate that representing epistatic interactions using BNs and scoring them using a BN scoring criteria holds promise for identifying epistatic relationships. Furthermore, they show that the use of the BDeu score with large values of α (54-162) can yield the best results on some data sets. Compared to MDR and other BN scoring criteria, these BDeu scores performed substantially better at detecting the hardest-to-detect models using simulated data sets, and at confirming previous results using a real GWAS data set.

## Methods

### Simulated Data Sets

Each simulated data set was developed from one of 70 epistasis models described in Velez et al. [10] (see Supplementary Table one in [10] for details of the 70 models). These datasets are available at http://discovery.dartmouth.edu/epistatic_data/.

Each model represents a probabilistic relationship in which two SNPs together are correlated with the disease, but neither SNP is individually predictive of disease. The relationships represent various degrees of penetrance, heritability, and minor allele frequency. The models are distributed uniformly among seven broad-sense heritabilities ranging from 0.01 to 0.40 (0.01, 0.025, 0.05, 0.10, 0.20, 0.30, and 0.40) and two minor allele frequencies (0.2 and 0.4).

Data sets were generated with case-control ratio (ratio of individuals with the disease to those without the disease) of 1:1. To create one data set they fixed the model. Based on the model, they then generated data concerning the two SNPs that were related to the disease in the model, 18 other unrelated SNPs, and the disease. For each of the 70 models, 100 data sets were generated for a total of 7000 data sets. This procedure was followed for data set sizes equal to 200, 400, 800, and 1600.

### GWAS Data Set

Several LOAD GWA studies have been conducted. We utilized data from one such study [16] that contains data on 312,316 SNPs. In this study, Reiman et al. investigated the association of SNPs separately in APOE *ε*4 carriers and in APOE *ε*4 noncarriers. A discovery cohort and two replication cohorts were used in the study. Within the discovery subgroup consisting of APOE *ε*4 carriers, 10 of the 25 SNPs exhibiting the greatest association with LOAD (contingency test *p*-value 9 × 10^-8 ^to 1 × 10^-7 ^) were located in the GRB-associated binding protein 2 (GAB2) gene on chromosome 11q14.1. Associations with LOAD for 6 of these SNPs were confirmed in the two replication cohorts. Combined data from all three cohorts exhibited significant association between LOAD and all 10 GAB2 SNPs. These 10 SNPs were not significantly associated with LOAD in the APOE *ε*4 noncarriers.

### Implementation

We implemented the methods for learning and scoring DDAGs using BN scoring criteria in the Java programming language. MDR v. mdr-2.0_beta_5 (available at http://www.epistasis.org) with its default settings (Cross-Validation Count = 10, Attribute Count Range = 1:4, Search Type = Exhaustive) was used to run MDR. All experiments were run on a 32-bit Server running Windows 2003 with a 2.33 GHz processor and 2.00 GB of RAM.

## Authors' contributions

XJ conceived the study, developed the DDAG model and the BNMBL score, conducted the experiments, and drafted the manuscript. RN identified the BN scores that were evaluated, performed the statistical analysis, and conceived and wrote Additional file 1. MB critically revised the manuscript for intellectual content concerning genetics. SV conceived the notion that we need not represent the relationships among SNPs, and critically revised the entire content of the manuscript. All authors read and approved the final manuscript.

## Supplementary Material

Additional file 1 **Illustrative Example of Better Large α Performance**. This file provides an illustrative example to demonstrate a possible explanation for the better performance of the BDeu score at larger values of α on hard-to-detect genetic models. Click here for file
