# Supplementary material for: Learning genetic epistasis using Bayesian network scoring criteria
Source: BMC Bioinformatics. 2011 Mar 31;12:89. doi: 10.1186/1471-2105-12-89 (PMC3080825; doi:10.1186/1471-2105-12-89)
Supplement: Additional file 1 — Illustrative Example of Better Large α Performance. This file provides an illustrative example to demonstrate a possible explanation for the better performance of the BDeu score at larger values of α on hard-to-detect genetic models. [file 1471-2105-12-89-S1.DOC]

# Appendix A

In this appendix we provide an illustrative example to demonstrate a possible explanation for the better performance of the BDeu score at larger values of α on hard-to-detect genetic models.

To score a DDAG with the Bayesian score we need only compute a term for the disease node, which has two states. So the Bayesian score for such a DAG is as follows:

$$\begin{matrix} score_{Bayes}(G : Data)= P(Data|G) \\ = \prod_{j=1}^{q} \frac{\Gamma(\sum_{k=1}^{2} a_{jk})}{\Gamma(\sum_{k=1}^{2} a_{jk}+\sum_{k=1}^{2} s_{jk})}\prod_{k=1}^{2} \frac{\Gamma(a_{jk}+s_{jk})}{\Gamma(a_{jk})}. \end{matrix}$$

Suppose that we are comparing two 2-SNP models in which each SNP has two states. In this case the number of parent states of the disease node is $q=4$. Suppose further that our data are as follows:

| Model | $s_{11}$ | $s_{12}$ | $s_{21}$ | $s_{22}$ | $s_{31}$ | $s_{32}$ | $s_{41}$ | $s_{42}$ |
| --- | --- | --- | --- | --- | --- | --- | --- | --- |
|  | 57 | 45 | 45 | 45 | 45 | 45 | 45 | 45 |
|  | 52 | 47 | 48 | 48 | 51 | 41 | 41 | 44 |

These are data we might obtain if $G_{1}$ is a hard-to-detect model that is generating the data, while $G_{2}$ is a distracter model which by chance exhibits a slight dependence between the disease node and the parent SNPs based on the data. We have the following scores for the two models:

$$=5. 80\times10^{-116}$$

$$=5. 90\times10^{-116}$$

$$=6. 15\times10^{-113}$$

$=6. 09\times10^{-113}$ .

Model $G_{2}$ scores highest when α = 2, but the correct model $G_{1}$ scores highest when α = 200. The larger value of α can attenuate the effect of smaller discrepancies between the number of disease cases and number of controls that occur by chance, leaving the largest discrepancy (namely 57/45) to dominate the result. The smaller value of α is not able to do this.

Suppose now our data is as follows:

| Model | $s_{11}$ | $s_{12}$ | $s_{21}$ | $s_{22}$ | $s_{31}$ | $s_{32}$ | $s_{41}$ | $s_{42}$ |
| --- | --- | --- | --- | --- | --- | --- | --- | --- |
| $G_{1}$ | 67 | 35 | 45 | 45 | 45 | 45 | 45 | 45 |
| $G_{2}$ | 54 | 47 | 50 | 42 | 53 | 43 | 45 | 40 |

These are data that we might obtain with an easier-to-detect model. In this case the scores for the two models are as follows:

$$score_{\alpha=2}(G_{1} : Data)=4. 51\times10^{-114}$$

$score_{\alpha=2}(G_{2} : Data)=2. 48\times10^{-116}$ .

Now $G_{1}$ wins easily even with α = 2 because the largest discrepancy (67/35) is so much larger than the other discrepancies.
